# Investigating the improvement of the quality of industrial effluents for reuse with added processes: coagulation, flocculation, multi-layer filter and UV

**DOI:** 10.1038/s41598-024-54310-7

**Published:** 2024-02-17

**Authors:** Razieh Esteki, Mohammad Hassan Ehrampoush, Habibeh Nasab, Ali Asghar Ebrahimi

**Affiliations:** Environmental Science and Technology Research Center, Department of Environmental Health Engineering, School of Public Health, Shahid Sadoughi University of Medical Sciences, Yazd, Iran

**Keywords:** Water reuse, Industrial wastewater, Coagulation and flocculation, Multi-layer filter, Ultraviolet rays, Environmental chemistry, Chemistry

## Abstract

Reuse of wastewater is one of the ways to develop water resources. In addition to the need for drinking water, many industries also need high-quality water in the production line. Therefore, the purpose of the present study is to investigate the advanced treatment of the wastewater treatment plant of Morche Khort industrial town using the processes of coagulation, flocculation with aeration, multi-layer filter, and disinfection by ultraviolet radiation to increase the quality of wastewater and reuse it in industries. In this study, to investigate the effect of coagulation and flocculation units along with aeration, filtration, and disinfection by ultraviolet rays (UV), on the quality of the secondary effluent from the wastewater treatment plant of Morche Khort industrial town, they were operated on a pilot scale. Polyaluminum chloride (PAC) was used as a coagulant. Layering of three layers of sand filter, from bottom to top including granulated silica at a height of 10 cm, sand at a height of 20 cm, and activated carbon at a height of 70 cm was used. The input and output sampling points of each unit were considered. By repeating twice in five stages of flow rates of 1, 2, 4, 6, and 8 (L/min), the samples were collected to determine COD, TSS, TDS, turbidity, pH, hardness, total coliform, and fecal coliform. Jar test results showed that Alum coagulant works almost the same as PAC in removing turbidity, but the efficiency of removing organic substances by PAC coagulant is higher than that of Alum at lower doses. The results of this study showed that the efficiency of the coagulation and flocculation process in removing turbidity, COD, TSS, TDS, and fat was 56.88%, 46.66%, 38%, 23.19%, and 91.43% respectively. In the current study, the results of the wastewater entering the sand filter showed that the percentage of removal efficiency with a loading rate of 1 (L/min) was turbidity, TSS, COD, TDS, and fat was 16. 93%, 56.84%, 50%, 5.67%, 33.44% respectively. In the UV disinfection unit, the removal efficiency percentage with a loading rate of 1 (L/min) for COD, TSS, turbidity, hardness, total coliform, and fecal coliform is 16%, 3.45%, 3.58%, 5.21%, 99.88%, and 98.37% respectively. Coagulation and flocculation system—multi-layer filter and disinfection can remove chemical–physical and microbial parameters to an acceptable level for using water in advanced purification systems and also for irrigation.

## Introduction

Currently, the water shortage has become a serious social and environmental challenge due to the increase in population, urbanization growth, public health, and expansion of industries and agriculture^1^. This is even though the water resources of the planet are almost constant the distribution of water in different regions is not the same and many places are facing water shortage^2^. For example, the Middle East region contains 6% of the world's population, while it contains only 1% of the world's freshwater resources^3^. The obvious fact is that conventional approaches to water supply in water-scarce areas are not sufficient^3^. Non-conventional water sources can be an alternative source of water and so overcome water scarcity and serve as an emerging opportunity to solve water resource constraints, especially in dry and semi-dry regions^1,4^.

One of the most important logical solutions for the development of water resources is the reuse of wastewater. These waters make up 65 to 80 percent of the water consumed by the communities, and due to the increase in the amount of water consumption per capita, it is increasing^5^. The development of new technologies has expanded the possibility of wastewater recovery. Recovered wastewater may have applications such as agriculture at all levels, irrigation of sports fields, urban and industrial uses, artificial feeding of underground water aquifers^3^.

Industrial wastewater management is essential to reduce health and socio-economic concerns^6^. The quantity and quality of industrial wastewater depend on the industrial process, raw materials, and products in each industrial unit; therefore, the composition of production effluents is different for any industry. For example, there are large amounts of salt, organic acids, and lignin in the wastewater of textile industries, chemical dyes in papermaking and pulp industries, in cosmetics and health industries there is a significant concentration of COD, oil, fat, and TSS^7^. conventional wastewater treatment does not meet minimum water quality standards for reuse, advanced treatment is required^8^.

Advanced wastewater treatment plays a very important and increasingly important role in urban and industrial wastewater treatment to achieve the quality goals of reused water and public health protection. Advanced treatment can be used to remove excess dissolved and suspended pollutants, nutrients, special metals, and other components of the wastewater. Common advanced treatment technologies include granular bed filtration, adsorption, membrane processes, and disinfection^9^.

Physicochemical treatment such as coagulation/flocculation is a suitable technique to reduce pollutants, especially colloidal particles and natural organic substances in wastewater^10^. The results of the study by Dafnopatidou, et al. showed that the use of coagulation and flocculation process along with aeration in textile factory wastewater treatment can reduce the amount of color by more than 97% and the possibility of reusing decolorized water is provided^11^. At present, the use of filtration methods for effluents from wastewater treatment processes has also become very common. Today, deep filtration is used to achieve the additional removal of suspended solids from the effluent of chemical and biological processes, as a pre-treatment for effective disinfection, and also as a pre-treatment step for membrane filtration^12^. The study of Bhutiani et al. showed that the use of a modified filter bed reactor (combination of sand and gravel) using sand intermittent filtration (SIF) is effective for removing various physicochemical parameters from industrial wastewater^13^.

Another treatment method to increase the quality of wastewater and reuse it is the use of surface absorption using activated carbon to absorb resistant organic compounds and the remaining amounts of inorganic compounds such as nitrogen, sulfides, and heavy metals, removing compounds It produces taste and smell. Under normal conditions, after treatment with activated carbon, the output BOD is in the range of 2–7 (mg/L) and the output COD is in the range of 10–20 (mg/L). Under optimal conditions, the outlet COD can be reduced to less than 10 (mg/L)^12^.

Disinfection of wastewater using chlorine leads to the formation of disinfection by-products that may be carcinogenic or have toxic effects on the consumer. Also, some pathogens are not easily inactivated by using chlorine. Therefore, an important effort was made in the world's water and wastewater treatment industry to find an alternative to chlorine^14^. The use of ultraviolet (UV) radiation for disinfection of reclaimed wastewater can lead to effective disinfection of bacteria, protozoa, and viruses in reclaimed wastewater^2,15^.

The studied industrial town (Morche Khort town) is located in an area with a dry climate. The amount of evaporation in this area is higher than the amount of rainfall. The underground water in this area is mainly located at a great depth of the earth, about 50 to 80 m from the surface of the earth. The maximum electrical conductivity is 5–6 millisiemens, which indicates the poor quality of well water and its low yield. Considering that 70% of the industries located in this town, in addition to the need for drinking water, also need good quality water in the production line, therefore, the purpose of this study is to investigate the advanced treatment of the wastewater treatment plant of Morche Khort industrial town using The processes of coagulation, chemical flocculation, along with aeration, multi-layer filter and disinfection by UV radiation are used to increase the quality of wastewater and reuse it in industries.

## Materials and methods

### The location of the study

Morche Khort industrial town is located 50 km northwest of Isfahan city, at longitude 51°28′ east and latitude 33°27′ north. This town is bordered by Mohammad Abad village from the north, Razavieh from the south, Alavijah from the west, and Rabat Sultan village from the east. Morche Khort industrial town has various industries such as chemical, metal, machinery, food, electricity and electronics, mineral, cellulose, and textile industries.

### Purification process

**Figure 1:**
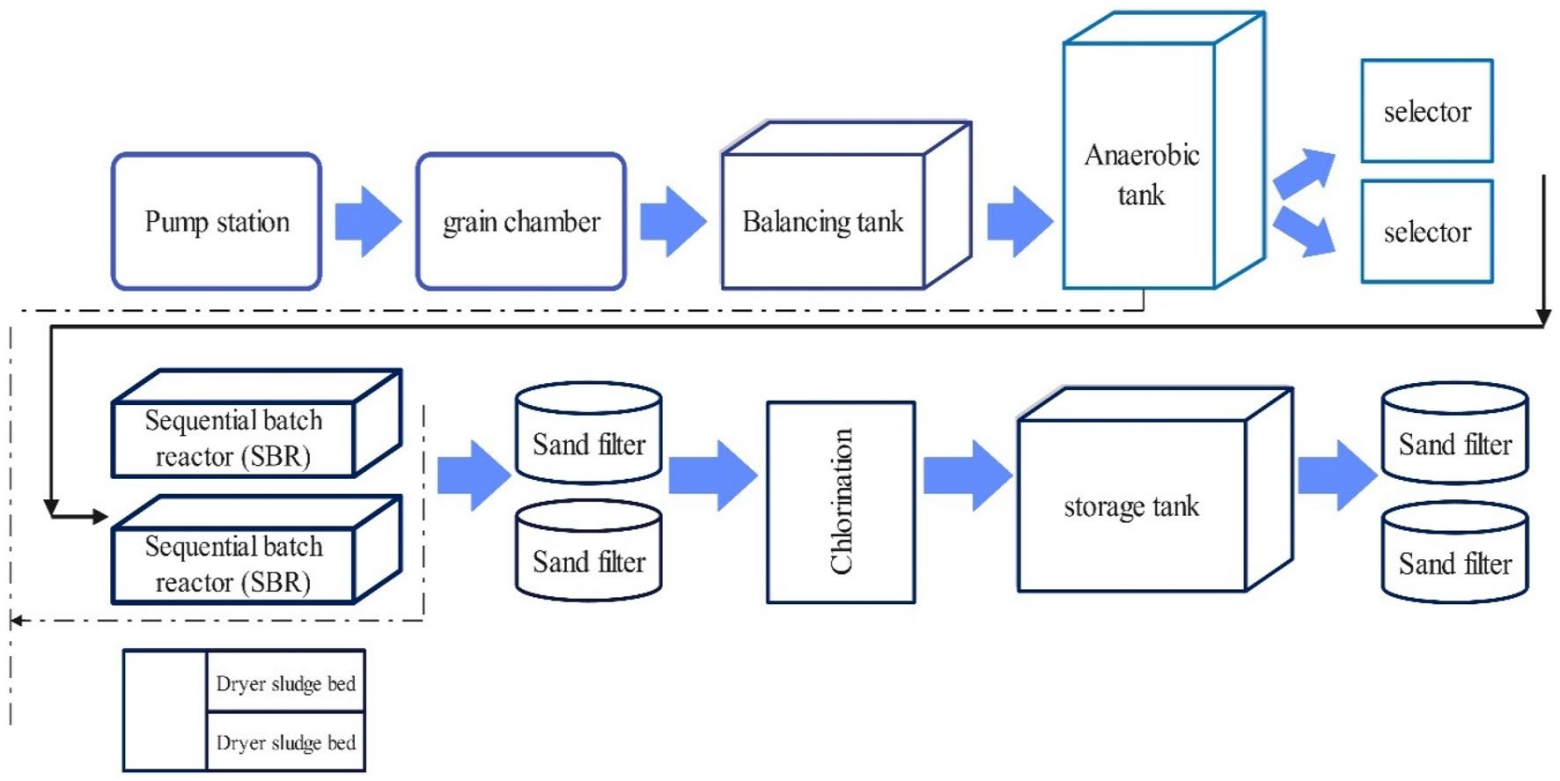
Schematic of the treatment plant of the studied industrial town.

The wastewater treatment process of Morche Khort industrial town currently consists of Screening, Grit Chamber, grease collection units, balancing and injecting materials to adjust the pH of the anaerobic contact pool, Sequence Batch Reactor (SBR) unit, and disinfection unit (chlorination) is. The schematic of the purification process is shown in Fig. 1.

### The process implemented in the present study

In this study, to investigate the effect of coagulation and flocculation units along with aeration, filtration, and disinfection by UV, on the quality of the secondary effluent from the wastewater treatment plant of Morche Khort industrial town, these units were operated on a pilot scale. The location of the pilot was considered before the storage and chlorination unit. The transfer of secondary wastewater to the pilot was done by installing a pump before the wastewater storage and chlorination unit^5^.

In the first stage of the process implemented in the pilot, polyaluminum chloride (PAC) was used as a coagulant (its optimal amount was determined using the Jar test) and it was injected by a pump at the point of entering the effluent into the tank (as a quick mix). The jar test was used to select the appropriate coagulant. At first, different coagulants including lime, PAC yellow, Chlorofric, PAC Solution, Alum, PAC Orange, PAC Orange, and Iron sulfate were compared in terms of turbidity and production sludge, and then two of the coagulants were selected (PAC and Alum). The average changes of effluent COD in different dosages of selected coagulants (PAC and alum) were compared to choose the best coagulant material. It should be noted that during the study, due to the variable quality of the effluent, the Jar test was repeated to inject the best dose. Then, coagulation-flocculation and sedimentation operations were used in a cylindrical tank consisting of two cylinders, one in the center of the tank for smooth mixing and the other cylinder with a larger size as a protective wall and improving the process of clot formation, also in this The tank was aerated using an aerating pump. After that, the treated effluent was discharged from the overflows and entered a sedimentation tank. Then the resulting effluent was pumped on a multi-layer sand filter. The layering of the sand filter consists of three layers, from bottom to top, including a layer of granular silica with a height of 10 cm, a layer of sand with a height of 20 cm, and a layer of granular activated carbon with a height of 70 cm took. Also, 15 cm of free space on the top of the bed was considered for the entry of sewage. Then, the effluent collected from the bottom of the filter was directed to the UV lamp for disinfection by a small valve and a very delicate hose^5,16^. The specifications of the UV lamp are given in Table 1.

**Table 1:** Specifications of the UV lamp used in the purification pilot.

Maximum operating pressure	125 (psi)	Flow	425 (MA)
The weight of the device	3.2 (kg)	Electricity consumption	14 (W)
Dimensions	D = 64 (mm) L = 340 (mm)	Maximum operating temperature	37 (C)
Input and output dimensions	3.8 (mm)	Number of bulbs	1
Voltage	110–120 (V)	The useful life of the lamp	9000 (h)
Frequency	50–60 (Hz)	The maximum radiation intensity in the flow rate	46 (mj/cm^2^)

### Sample volume

**Figure 2:**
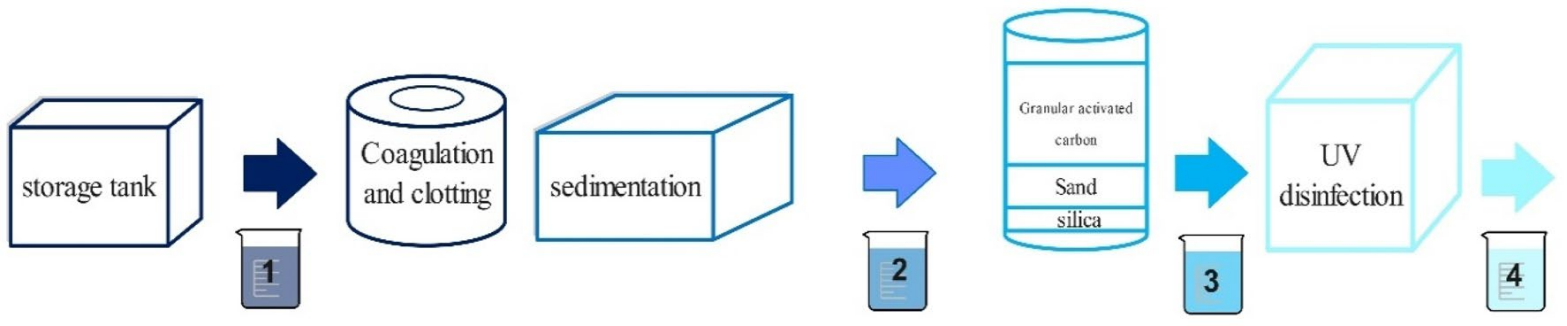
Schematic of the purification process in the studied pilot.

During the period of conducting the present study, the study pilot was intermittently used and sampled. Samples to determine amount of COD (mg/L), TSS (mg/L), TDS (mg/L), turbidity (NTU), EC(µSiemens/cm), pH, Hardship (mg/L), total coliform (MPN/100mL), fecal coliform (MPN/100mL), fat and oil (mg/L) with 2 repetitions from four places during five stages of flow rates of 1, 2, 4, 6, 8 (L/min) was gathered. The tests of physical, chemical, and biological variables were performed based on standard methods^16^. Location one includes the secondary treated effluent from the industrial town treatment plant or the effluent entering the coagulation and flocculation unit, location two is the effluent from the coagulation and flocculation unit, location three is the effluent from the multi-layer sand filter and the fourth location is the effluent from the disinfection unit was considered. Figure 2 shows the schematic of the study pilot.

### Optimal range of water consumption indicators in industries

Since the water consumption in each industry is different and each industry usually needs several types of water with different qualities, therefore, according to the indicators, the water consumption in the industries can be calculated according to the water standard of the industrial cities of Iran. Generally classified into 4 categories. This division is based on the required water quality and the amount of purification required to achieve the desired quality. In each group, the general and specific uses of different industries are specified. General uses include coolers, boilers, sanitary uses, material transfer, air conditioning, irrigation, and surface washing. which is often common in all industries. It should be noted that this classification is general and the quality range specified for each indicator may be the closest possible to the limit specified for the indicators by the water requirements of the processes mentioned in that category. In the case of a more detailed classification, the number of industrial water groups will reach many times. To avoid increasing the number of groups, the closest possible mode is considered. Table 2 shows the optimal range of industrial water indicators of different groups^17,18^.

**Table 2:** Optimal range (mg/liter) of water consumption indicators in industries.

Variable	First group	The second group	The third group	The fourth group
Fe (mg/L)	0–0.05	0–0.1	0–0.3	0–1
Mn (mg/L)	0–0.01	0–0.05	0–0.3	0–1
pH	7–9	6–10	5–10	5–10
COD (mg/L)	0–5	0–10	0–20	0–75
Hardship (mg/L)	0–1	0–100	0–250	0–500
Alkalinity (mg/L)	0–50	0–75	0–150	0–500
Sulphate (mg/L)	0–20	0–100	0–250	0–500
Silica (mg/L)	0–5	0–10	0–20	0–50
Turbidity (NTU)	0–1	0–5	0–10	0–100
TDS (mg/L)	0–50	0–100	0–500	0–1000
Cl^−^ (mg/L)	0–20	0–100	0–200	0–500

## Results and discussion

### Coagulation processes, chemical flocculation with aeration

In surface water treatment, the coagulation process is one of the most common processes used in different dimensions. The main purpose of using this process is to improve the efficiency of removing small particles that cause turbidity in the sedimentation pond, after which filtration is used to smooth and clarify the water. It is a process efficiency index. In recent years, in addition to using the coagulation process to remove turbidity (NTU), it has also been used to remove other organic and inorganic pollutants from raw water and wastewater. Table 3 shows the results of the jar test to select the best coagulant among several coagulants. The results of the TSS (mg/L), COD (mg/L) removal efficiency in the coagulation-flocculation and sedimentation stage by the application of soluble PAC coagulant in the present study (Table 3) showed that particles of different sizes were removed in colloidal and suspended form, as well as organic substances.

**Table 3:** Jar test results to determine the appropriate coagulant.

Variables	Raw sample	Lime	PAC yellow	Chlorofric	PAC solution	Alum	Alum	PAC orange	PAC orange	Iron sulfate
Amount (g/L)	–	0.3	0.3	0.3	0.3	0.3	0.5	0.3	0.5	0.3
Turbidity (NTU)	79	429	41	101	101	25.6	11.3	32	15.1	589
Amount of settled sludge (mL/L)	–	35	95	60	85	80	100	90	180	15

Table 4 shows the comparison results of Alum and PAC in removing COD (mg/L). The results showed that Alum coagulant also works almost the same as PAC in removing turbidity (NTU), but more efficient in removing organic substances was observed by PAC coagulant at lower doses than Alum. Also, the effect of this coagulant was observed in times when the quality of the effluent from the biological treatment system was not very favorable and it had suspended substances and higher organic load than normal conditions.

**Table 4:** The average changes of effluent COD in different doses of coagulant in the jar test.

Variables	Amount of coagulant (mg/L)								
	0	0.03	0.05	0.07	0.1	0.15	0.2	0.25	0.3
Alum (mg/L)	69.2	61.2	50.8	49.65	48.5	46.7	44	40.1	56.7
PAC (mg/L)	69.5	62.3	52.7	51	44.4	39.5	37.6	42.2	44.7

Table 5 shows the specifications of the incoming and outgoing effluent from the coagulation and flocculation unit. The present study showed that the average efficiency of the coagulation and flocculation process in removing physical and chemical parameters including turbidity (NTU), COD, TSS, TDS, and fat (mg/L) was 56.88%, 46.66%, 38%, 23.19%, and 91.43% respectively. (Table 5). So far, several types of research have been conducted in the field of application of coagulation and flocculation processes regarding the reuse of wastewater. Some of these studies have used sedimentation coagulation and others have used in-line coagulation before Ultrafiltration (UF), that is, the use of coagulant without removing solids before UF, and the results of some studies conflict with the results of other studies. For example, Dempsey et al. used coagulation in the pre-UF line and the results of their study showed that the coagulation conditions were unsuitable for conventional treatment, especially the low dose of coagulant that produced particles that could neither settle nor be removed in the Sand filter, these conditions are effective in coagulation in the pre-UF line and improve the removal of pollutants and improve the hydraulic performance of the filter^19^. In contrast to the study of Dialynas et al., they investigated the effect of coagulation in the line before UF, the results of their study showed that coagulation in the line and ultrafiltration did not have a significant effect on the removal efficiency of COD (mg/L), turbidity (NTU) and coliform (MPN/100 mL) parameters compared to direct UF of the effluent. They interpreted the reason as coagulation caused the removal of a part of the colloidal material that was larger than the UF pore size and had no effect on the removal efficiency^20^. Also, in a study conducted by Os-Masuda et al. in Nigeria, it was shown that the use of coagulation and flocculation process using chlorophric coagulant and polyelectrolyte coagulant will have the best effect for industrial wastewater treatment^21^.

**Table 5:** Specifications of incoming and outgoing effluent from coagulation and flocculation unit.

Variables	N	Inlet effluent			Coagulation		
		Mean	Min	Max	Mean	Min	Max
Turbidity (NTU)	4	4.35	4.2	4.5	1.96	1.94	1.98
pH	4	7.67	7.54	7.81	8.39	8.33	8.45
EC (µSiemens/cm)	4	6.75	6.66	6.91	5.28	5.13	5.43
TDS (mg/L)	4	67	65	69	54	53	55
TSS (mg/L)	4	46.5	43	50	32.5	31	34
COD (mg/L)	4	44	43	45	25	24	26
Fat and oil (mg/L)	4	7	7	8	0.6	0.5	0.7

### Using multi-layer filter

The filtration process with granular media as an advanced treatment method is mainly used to remove suspended particles from wastewater. The first application of granular media filtration in wastewater treatment is essentially a sequence of design methods used in drinking water treatment. Because the physical and chemical characteristics of wastewater are significantly different from most natural waters, its purification requires special design considerations. In general, sewage filters receive larger, heavier, and more diverse grains, and their solids loading is non-uniform. Filtering mechanisms are complex and may be a combination of factors such as compression (mechanical and random contact), screening, gravity settling, compression due to static pressure of particles along with sticking to the filter media, and the growth of biological solids in the filter bed. It causes more separation of solid materials. Because the performance of sewage filters is influenced by various factors, especially the location of the treatment plant, in cases where it is necessary to comply with the permitted output limits, semi-industrial unit studies are recommended.

Table 6 shows the characteristics of the effluent from the multi-layer sand filter unit at different flow rates. The results showed that the removal efficiency percentage of physical and chemical parameters with a loading rate of 1 (L/min) in the multi-layer sand filter was turbidity (NTU), TSS, COD, TDS, and fat (mg/L) were 16. 93%, 56.84%, 50%, 5.67%, 33.44% respectively. With the loading rate of 2 (L/min) were 90%, 54.84%, 54.17%, 5.67%, and 25% respectively. With a loading rate of 4 (L/min) were 88.69%, 40.33%, 50%, 3.78%, and 25%, respectively. With a loading rate of 6 (L/min) were 88.16%, 40.33%, 43.75%, 2.84%, and 25% respectively. With a loading rate of 8 (L/min) were 88.16%, 40.33%, 43.75%, 2.84%, and 25%, respectively (Table 6).

**Table 6:** Specifications of the effluent from the multi-layer sand filter.

Variables	N	1 (L/min)			2 (L/min)			4 (L/min)			6 (L/min)			8 (L/min)		
		mean	Min	Max	mean	Min	Max	mean	Min	Max	mean	Min	Max	mean	Min	Max
Turbidity (NTU)	4	0.14	0.13	0.15	0.19	0.19	0.20	0.21	0.21	0.22	0.22	0.22	0.23	0.22	0.22	0.23
pH	4	8.47	8.45	8.49	8.46	8.42	8.50	8.44	8.43	8.46	8.45	8.45	8.46	8.46	8.45	8.48
EC (µSiemens/cm)	4	5.13	5.13	5.14	5.17	5.15	5.20	5.17	5.17	5.17	5.17	5.17	5.17	0.17	5.17	5.18
TDS (mg/L)	4	50.5	50	51	51	50	52	51	51	51	51.5	51	52	51.5	51	52
TSS (mg/L)	4	14.5	14	15	14.5	14	15	18.5	18	19	18.5	18	19	18	17	19
COD (mg/L)	4	12.5	12	13	11.5	11	12	12	11	13	13.5	13	14	13.5	13	14
Hardship (mg/L)	2	365	350	380	365	350	380	365	350	380	365	350	380	365	350	380
Total coliform (MPN/100mL)	2	1.6 × 10^5^	1.7 × 10^5^	1.2 × 10^4^		1.5 × 10^5^	1.5 × 10^5^		1.5 × 10^6^	1.5 × 10^6^		1.6 × 10^5^	1.6 × 10^5^		1.5 × 10^5^	1.6 × 10^5^
Fecal coliform (MPN/100mL)	2		1.5 × 10^5^	1.09 × 10^4^		1.4 × 10^4^	1.5 × 10^4^		1.5 × 10^4^	1.6 × 10^4^		1.5 × 10^4^	1.5 × 10^4^		1.5 × 10^5^	1.6 × 10^5^
Fat and oil (mg/L)	4	0.4	0.3	0.5	0.45	0.4	0.5	0.45	0.4	0.5	0.45	0.4	0.5	0.45	0.4	0.5

So far, several types of research have been conducted in the field of various pre-treatment methods to remove these substances from wastewater, and various techniques such as sand filtration, coagulation, and active carbon absorption have been studied. In Zheng et al.'s study, the effect of a slow sand filter on the removal of total dissolved solids from wastewater was investigated. The results showed that the percentage of removal efficiency was 10%, 27%, and 34%, respectively, for proteins, polysaccharides, and biopolymers at a loading of 0.25 (m/h)^22^. The results of the present study clearly showed that the multi-layer sand filter does not have a significant effect on TDS (mg/L) removal, because, in all 5 loading ranges, the highest removal rate of about 4% in TDS (mg/L) was observed. The amount of organic matter removal that has been achieved in the form of COD (mg/L) and turbidity (NTU) reduction can be caused by the removal of suspended and colloidal organic matter.

### Disinfection by UV

Table 7 shows the characteristics of the effluent from the UV unit at different flow rates. The results showed the percentage of removal efficiency with a loading rate of 1 (L/min) in the disinfection unit with UV rays for COD (mg/L), TSS (mg/L), hardness (mg/L), turbidity (NTU), total coliform (MPN/100mL), and fecal coliform (MPN/100mL) were 16%, 3.45%, 3.58%, 5.21%, 99.88%, and 98.37% respectively. The percentage of removal efficiency with a loading rate of 2 (L/min) were 8.7%, 28.21%, 4.94%, 99.88%, 98.8%, and 98.37% respectively. The percentage of removal efficiency with the loading of 4 (L/min) were 12.5%, 24.43%, 6.98%, 99.88%, 98.84%, and 98.37% respectively. The percentage of removal efficiency with the loading of 6 (L/min) were 11.12%, 24.43%, 11.12%, 99.89%, 98.7%, and 98.37% respectively. The percentage of removal efficiency with a loading rate of 8 (L/min) were 11.12%, 22.23%, 11.12%, 4.66%, 99.89%, and 17.98% respectively. The results of the present study showed that UV has a high efficiency in removing total coliform and fecal coliform from wastewater treatment plants, which has an important effect on advanced wastewater treatment.

**Table 7:** Characteristics of UV unit effluent.

Variables	N	1 (L/min)			2 (L/min)			4 (L/min)			6 (L/min)			8 (L/min)		
		mean	Min	Max	mean	Min	Max	mean	Min	Max	mean	Min	Max	mean	Min	Max
COD (mg/L)	4	10.5	10	11	10.5	10	11	10.5	10	11	12	12	12	12	12	12
TSS (mg/L)	4	14	14	14	14	14	14	14	14	14	14	14	14	14	14	14
Turbidity (NTU)	4	0.13	0.13	0.14	0.14	0.3	0.15	0.20	0.20	0.20	0.20	0.20	0.20	0.20	0.20	0.20
pH	4	8.36	8.35	8.37	8.30	8.30	8.31	8.33	8.31	8.35	8.33	8.31	8.35	8.34	8.32	8.36
Hardship (mg/L)	2	346	342	350	347	342	352	347	342	352	346.5	341	352	348	344	352
Total coliform (MPN/100mL)	2	1.8 × 10^2^	1.7 × 10^2^	1.9 × 10^2^	1.8 × 10^2^	1.8 × 10^2^	1.9 × 10^2^	1.8 × 10^2^	1.8 × 10^2^	1.9 × 10^2^	1.8 × 10^2^	1.8 × 10^2^	1.9 × 10^2^	1.8 × 10^2^	1.8 × 10^2^	10^2^ × 2
Fecal coliform (MPN/100mL)	2	–	1.8 × 10^2^	1.8 × 10^2^	1.8 × 10^2^	1.8 × 10^2^	1.8 × 10^2^	1.8 × 10^2^	1.8 × 10^2^	1.8 × 10^2^	1.8 × 10^2^	1.8 × 10^2^	1.8 × 10^2^	1.8 × 10^2^	1.8 × 10^2^	1.8 × 10^2^

The results of Amin et al.'s study by measuring the amount of fecal and total coliform, fecal streptococcus (MPN/100mL), TSS, iron, and hardness (mg/L) to check the performance of the clarification unit and UV in the treatment and disinfection of sewage treatment plant effluent, showed improvement in the penetration of UV rays due to sedimentation. For coarse particles and clots, wastewater disinfection with a relatively high irradiation time is possible in common doses, although to improve the quality of the wastewater and disinfect the flow rate, it is recommended to use an advanced purification system such as filtration before installing the UV lamps^14^. The results of the study by O-Mi Lee et al. showed that for the disinfection of the effluent from secondary treatment in the wastewater treatment plant, three types of disinfection were used separately: UV, ozone, and ion radiation for a removal rate of 99.9% (log1) 93.53, 36.80 (l.day) whare needed, respectively, which means that the ion radiation method is more economical^23^.

## Conclusion

- The studied treatment system can remove coliform bacteria to the extent of meeting the environmental standards of the wastewater in the treatment plant of Morche-Khort Industrial Town.
- The studied treatment system is efficient in removing electrical conductivity and inefficient dissolved organic matter and in removing turbidity, TSS, microbial load, and COD, especially since it has a reduction of more than 90% in the turbidity of the incoming effluent.
- Coagulant injection has increased the removal efficiency of study parameters.
- The coagulation and flocculation system combined with aeration is efficient in removing fat and oil.

## Data Availability

The supporting data are available from the corresponding authors upon reasonable request.
